# Transboundary movement of waste review: From binary towards a contextual framing

**DOI:** 10.1177/0734242X221105424

**Published:** 2022-06-22

**Authors:** Kaustubh Thapa, Walter JV Vermeulen, Pauline Deutz, Olawale E Olayide

**Affiliations:** 1 Copernicus Institute of Sustainable Development, Utrecht University, Utrecht, The Netherlands; 2 Department of Geography, Geology and Environment, University of Hull, Hull, UK; 3 Department of Sustainability Studies, University of Ibadan, Ibadan, Nigeria

**Keywords:** Transboundary waste movement, Basel Convention, international waste flow, waste trade, waste

## Abstract

Multiple cases of toxic waste dumping from the Organisation for Economic Co-operation and Development (OECD) countries to non-OECD countries in the 1980s led to scholarly attention to transboundary waste movements. The Basel Convention was established to provide an international legal framework to tackle such problems in the early 1990s, focusing on hazardous waste. However, the transboundary movement of all waste, not just hazardous waste, remains a societal challenge globally, frequently surfacing as an ethical question on the one hand and a story of resource management/trade on the other. This phenomenon has been studied across disciplines resulting in diverse, scattered and often contested understandings. Despite previous and ongoing efforts, waste production, management and transboundary movements are increasing and are predicted to grow significantly with global social, environmental and economic implications. This literature review uses a research synthesis and problematisation approach to critically analyse the transboundary waste literature since 1985. The findings highlight research trends, the need for data reliability and policy coherence, and the sustainability implications of the phenomenon. One recurring theme in the literature is the reduction of the complex phenomenon involving multiple countries, policies, actors and waste streams into simple opposite narratives, which we called transboundary waste binaries. We have identified and then challenged assumptions behind transboundary waste binaries and discussed the implications of such assumptions on the broader discourse. We have concluded with future research recommendations to look past the transboundary waste binaries towards a nuanced and contextual understanding of transboundary waste flows.

## Introduction

Like commodities, waste is imported and exported from one country to another (Cotta, 2020; Kellenberg, 2012; Lipman, 2015). This practice has social, environmental and economic implications and is a sustainability challenge at a global level (Cotta, 2020; Yang, 2020). The transboundary movement of waste consists of various waste streams governed by a patchwork of national, regional and international laws. United Nations Environment Programme (UNEP) estimates that 11.2 billion tonnes of solid waste is collected globally every year and estimates the global waste market sector, from collection to recycling, to be worth US$ 410 billion (UNEP, 2012, 2020). This estimate excludes the value added by the informal sector, which accounts for 80% of the waste recycling in low-income countries (Rucevska et al., 2015). There is currently no reliable estimate for the amount of all waste categories that cross international boundaries for disposal. Notably, countries that lack the capacity for sound management of their own waste are included among the destinations of transboundary waste exports. More than half of the global population depends on active dumpsites for disposal, predominantly in low-income countries, posing health and environmental risk (UNEP, 2015). Waste generation and disposal contribute 5% of the global greenhouse emissions, and sound waste management links to 12 of the 17 Sustainable Development Goals (UNEP, 2015).

Everyday human actions create various waste streams, some of which move internationally. Understanding the transboundary waste movement involves first understanding waste as a concept with varied socio-cultural, political and economic interpretations. Douglas (1966) argued that interpretations of waste are based on the cultural context of social classifications and their relationships, coining the phrase: ‘dirt is matter out of place’. Reno attributed waste as ‘a mirror of human culture’ and as the ‘signs of life’ (Reno, 2014). Waste perceptions encompass deep cultural norms shaped by products, materials and contexts and waste is often stigmatised in many societies (van Ewijk et al., 2018). Waste ‘out of sight’ is often ‘out of mind’ and usually is desirable elsewhere but ‘not-in-my-backyard’. UNEP remains vague about defining waste in their *Global Waste Management Outlook* report, acknowledging various usages of the word and views waste as ‘the combination of four wrongs - a wrong substance, in a wrong quality, in a wrong place at a wrong time’ and later refers to the colloquial understanding as ‘stuff people throw away’ (UNEP, 2015).

Like UNEP’s the Basel Convention on the Control of Transboundary Movement of Hazardous Waste and its Disposaland showed their limitations leaves ample room for interpretation, thereby creating confusion. It defines waste as ‘substances or objects which are disposed of or are intended to be disposed of or are required to be disposed of by the provisions of national law’ (The Basel Convention, 1992). Despite, or because, waste is essentially something that someone does not want, a legal definition is required to identify substances that need to be handled in specific ways to prevent harm to health or the environment. Sound waste management imposes costs, creating an incentive to seek an illegal solution, and the legal definition retains the contingent nature of the informal definitions. Discarded tyres for safety standards in one place might become second-hand tyres elsewhere with less stringent laws and then disposed of without guaranteeing sustainability (Campbell-Johnston et al., 2020). Like other waste, discarded tyres can either be recycled using sophisticated technology, incinerated, dumped or landfilled (locally or internationally), or used as secondary resources depending on the local socio-economic and environmental context. The potential for diverse interpretations of waste (even in cases with established legal definitions) is magnified more when waste moves from one national jurisdiction to another. Waste in one place can even be second-hand goods in others (such as discarded clothes or even a battleship) or raw material (such as plastic, paper and vehicle waste). Given the subjectivity of what is worth or safe to reuse and the capacity for reuse as raw material, there is a blurred line between trade in second-hand goods and a trade explicitly in waste. The same substance can be different things to different people and change its legal status in transit based on our assumptions of waste. This ambiguity of the waste definitions leading to multiple interpretations of waste has policy implications, influencing its sustainability implications (see section ‘Policy gaps’).

Among various socio-economic and political factors, the differences in disposal or recycling costs between places can drive the transboundary movement of waste with or without any regard for its sustainable management. A substance or item can become waste (which needs to be disposed of), end-of-life (which requires either to be disposed of or to be brought back to life) or resource (which is valorised) depending on its context. The processes applied (e.g. disposal or valorisation) are knowledge and technology-dependent, which depend on socio-cultural, political and economic realities. Moreover, something potentially hazardous can be treated without harm given appropriate technology and regulatory control, whereas it can cause harm elsewhere in the absence of one or the other. The identification of waste then is open to different interpretations in varied social, economic, political and cultural contexts. These diverse contexts and interpretations become even more pronounced when factoring in various actors and their worldviews engaged in the transboundary waste movement.

Academic scholarship in the transboundary movement of waste started in the 1980s following some notorious cases of hazardous or toxic waste dumps by the Organisation for Economic Co-operation and Development (OECD) countries to the non-OECD countries (Clapp, 2001). The Basel Convention entered into force in 1992 and remains the foremost multilateral agreement governing transboundary hazardous waste. The three aims and provisions of the Basel Convention, when it entered into force in 1992, were: ‘the reduction of hazardous waste generation and the promotion of environmentally sound management of hazardous wastes, wherever the place of disposal’; ‘the restriction of transboundary movements of hazardous wastes except where it is perceived to be in accordance with the principles of environmentally sound management’ and ‘a regulatory system applying to cases where transboundary movements are permissible’. (The Basel Convention, 2011). Since the Basel Convention entered into force, volumes of hazardous waste generation have increased significantly and are predicted to increase further (The World Counts, 2021). Its transboundary movement remains an ongoing challenge (The Basel Convention, 2011). Overcoming the challenges is also technology and capital intensive (Yang, 2020). It is worth noting that the Basel Convention only covers the transboundary movement of hazardous waste, which comprises a significant and important fraction of all waste. Non-hazardous wastes, such as textile, paper, etc., are openly traded globally as commodities following the World Trade Organization (WTO) rules (Grosz, 2016). Sound management of all forms of waste, hazardous or non-hazardous, and its transboundary remains a societal challenge. Even with several policy gaps (see section ‘Policy gaps’), the Basel framework is the key international regulatory tool critiqued and debated in the literature.

This article reviews and critically analyses the existing transboundary movement of waste (both hazardous and non-hazardous waste) literature from 1985 to 2021. While some literature reviews on specific waste streams consider transboundary waste flows (see Fazzo et al., 2017; Maphosa et al., 2020; Pérez-Belis et al., 2014), a recent review dedicated to transboundary waste movements is lacking. We present an integrated review of the topic spanning the last 35 years and then identified the most prevalent assumptions in the literature. We used the ‘problematisation approach’ (Alvesson and Sandberg, 2011) to challenge some existing discourses and their implications. To problematise, we asked ourselves: what are the core assumptions in the discourse? Can we challenge these core assumptions to create helpful problem-solving knowledge moving forward? This integration and problematisation of existing knowledge in this review can help future researchers frame their research to generate more contextual problem-solving knowledge, which might then translate to problem-solving policymaking and actions. This article first discusses literature review methods. We present findings showing scholarship trends, map geographical areas of academic publications, highlight data scarcity and policy gaps and established linkages of transboundary waste movement to sustainability. In section ‘Analysis and discussion: Transboundary waste binaries’, we focuse on the concept of waste as both a resource and discard and examined the existing binary-dominated understanding (e.g. waste/not waste; legal/illegal). We explain these transboundary waste binaries, challenged some of their built-in assumptions and showed their limitations. We then recent work examining transboundary waste movements that consider the political, cultural, social and economic context and challenge these transboundary waste binaries. We highlight a need for a more contextual understanding of the transboundary movement of waste moving forward. Based on the findings and discussion, we conclude with five future research suggestions.

## Methods

This section presents the frameworks used in this literature review, the literature review process and the limitations of the study.

### Review methods

In this research, we used a combination of two frameworks. For the literature review, we used the framework developed by Cooper (2016) for research synthesis. The framework enables us to look at multiple studies on the same topic and draw a conclusion about the research findings. Research synthesis includes the following steps: formulating the problem, searching the literature, gathering information from studies, analysing, integrating the outcome of the studies, interpreting the evidence and presenting the result (Cooper, 2016). We integrated previous works, sometimes by comparing and contrasting, and other times connecting the literature. After this, we used the ‘problematisation approach’ of Alvesson and Sandberg (2011) for a critical analysis of the findings. This approach focuses particularly on spotting assumptions. The problematisation approach thus enables us to re-evaluate existing understandings and reimagine our ways of thinking about them. In the past, the primary focus of waste management was limited to collection and recycling. Now, there is a need to reduce and valorise waste – even before thinking of recycling. This view-shift, drawing on the principles of the circular economy (Stahel, 2019), calls for re-examining ideas that shaped policies and practices, which had real-world implications. We utilised the problematisation method to spot assumptions reflexively, question them and explore the possibility of thinking differently. We analysed a set of similar assumptions in the literature, which we call transboundary waste binaries.

Given the diverse and context-dependent interpretations of waste and its transboundary movement, these two methods enable a new and integrated contextual understanding. Research synthesis primarily guides the result section (see section ‘Results’), and the problematisation approach guides the analysis (and discussion) section (see section ‘Analysis and discussion: Transboundary waste binaries’).

### Literature review process

The literature review process includes three phases: searching, filtering and data collecting. The first phase of the research involved searching academic literature for keywords and their combination in the Scopus database for papers published between January 1985 and January 2021. These keywords include ‘waste’, ‘export’, ‘international’, ‘flow’, ‘movement’, ‘trade’, ‘transboundary’, ‘waste’, and ‘hazard’, which were then refined by the phrases: ‘waste flow’, ‘waste trade’, ‘waste movement’, ‘international waste’, ‘transboundary waste’, ‘global waste’, ‘transboundary movement’, ‘waste export’ and ‘transboundary waste movement’. These terms, phrases, and their combinations (inclusion criteria) were searched using the Boolean operators ‘and’, ‘or’ and ‘and not’ in 10 different searches that provided articles relevant to the transboundary waste movement. This process resulted in 1907 relevant articles, which were saved with their abstracts.

**Figure 1.**
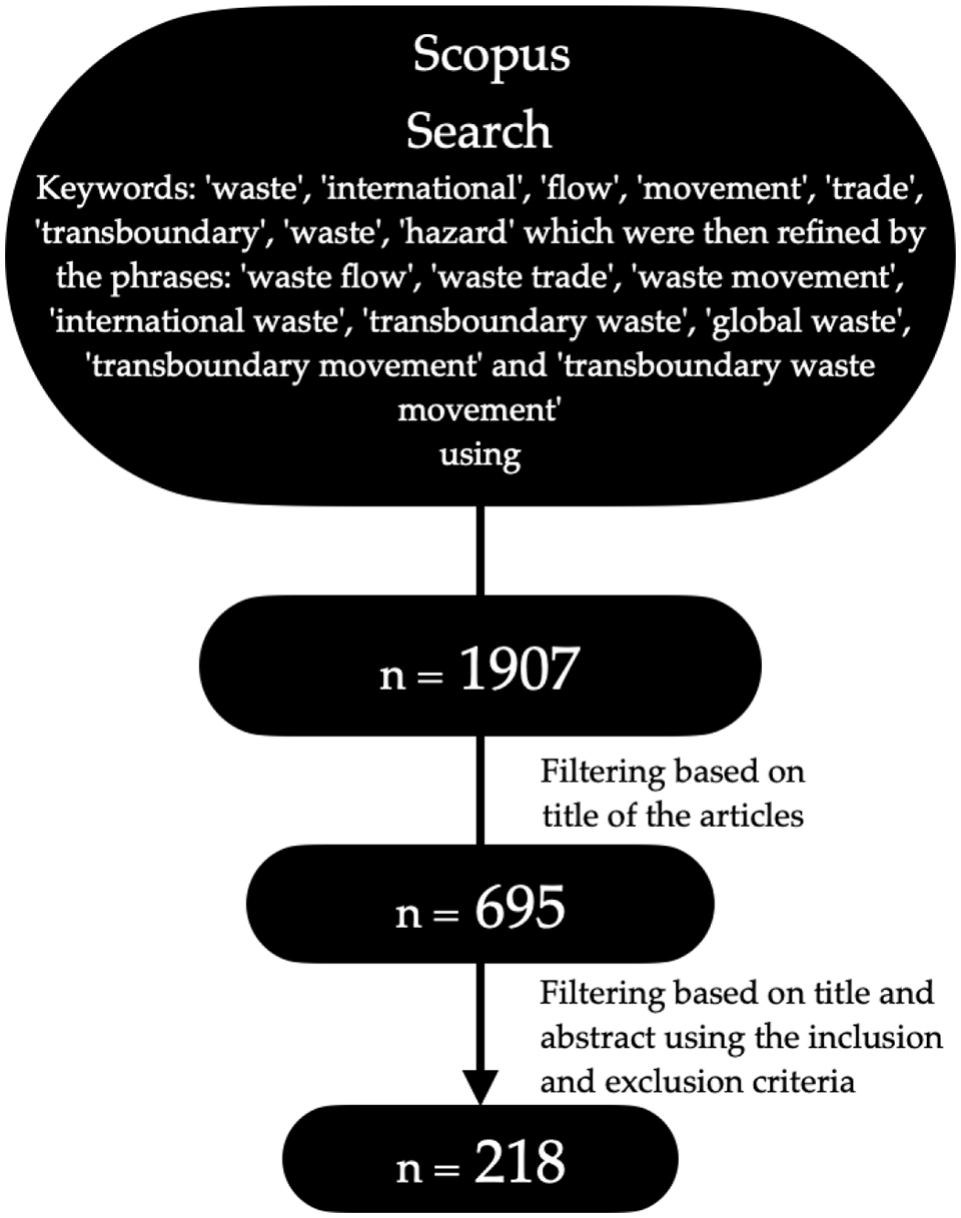
The process of screening and filtering used in this study to reach 218 articles from January 1985 to January 2021.

The second phase of screening and filtering followed the three-step process illustrated in Figure 1. Firstly, the title of the journal articles or book chapters from the 1907 articles were screened using keywords. Where the titles provided insufficient information, their abstracts were analysed. This filtering process resulted in narrowing it down to 695 articles. The next step was to analyse the abstracts of the articles and book chapters, applying excluding criteria. As the transboundary movement of solid waste is the subject of this research, transboundary waste movement of air or water pollution was excluded. Publications that did not focus on the transboundary movement of waste as the primary subject were also excluded. For instance, if a paper focused on improving techniques for ship recycling and mentioned transboundary movements of end-of-life ships only briefly, the paper was discarded. In some cases, where the information in the abstract was not sufficient, the paper was scanned with attention to the above-identified keywords, and irrelevant articles were excluded. The process narrowed down the sample size to 218 articles. For this review, we analysed 218 full texts on the transboundary movement of waste between January 1985 and January 2021.

As most of the resulting articles focus on the transboundary movement of waste from OECD countries to non-OECD countries, this category of transboundary movement is the subject of this research. The academic focus on OECD and non-OECD countries most likely reflects the formulation of the Basel Convention itself. At the same time, other literature works with the Global North and South^1^ terminology. There are important flows of transboundary waste within the countries in the Global North (see Bisschop, 2012; Obradović et al., 2014; O’Neill, 1997; Thomas and Fannin, 2011), within Global South (see Lepawsky, 2015a; Shinkuma and Huong, 2009) or from the countries in Global South to Global North, which are only discussed briefly in the literature and need more attention. The problems brought about by hazardous waste movement between economically and geographically distinct categories of countries, as tackled by the Basel Convention, remain.

### Limitations

Before presenting the findings, it is important to discuss the limitations of the research. Firstly, as established in the introduction, understanding of waste is shaped by a socio-cultural vantage point, which inherently leads to biases of the researchers. Analysing the articles for the discussion section involved selecting issues to problematise, reflecting the authors’ socio-cultural vantage point. For instance, the social scientist using a colonial lens might problematise the lack of decolonising discourse and note specific relevant elements. Precautions were taken to incorporate different points of view and across academic disciplines by authors from three institutions with diverse academic backgrounds working in sustainability research. Secondly, only Scopus, a widely used database among social and natural scientists, was selected; incorporating other similar databases might have increased the number of articles analysed. Based on other similar literature reviews, 218 articles are deemed sufficient to capture the variety of discussions on the transboundary movement of waste. Non-academic sources, such as reports, whitepapers, documentaries and news articles, are not included as our purpose is to review academic perspectives. However, 36 years of academic publications include various relevant vantage points of understanding, including waste exporting/importing countries, the policy community, NGO, activist community, etc. Thirdly, the search keywords resulted in articles framed in the context of or relevant to transboundary waste movements. However, it could exclude broader waste literature (prevention, reduction, recycling, etc.) but not explicitly linked to the transboundary movement. Lastly, only English language scientific publications were chosen, the majority of which are from institutions in OECD countries. Although authors of those papers might be based in or originating from non-OECD countries, the resulting literature on transboundary waste might not reflect a globally diverse socio-cultural framing or a plurality of understanding.

## Results

Firstly, we presented and discussed the research trends around the various waste streams and geographies of research. Secondly, we found that data scarcity and policy gaps remained a recurrent theme in the literature. Thirdly, we presented sustainability implications of the transboundary movement of waste and the lack of discourse on reducing transboundary movement found in the literature.

### Trends and waste categories

**Figure 2.**
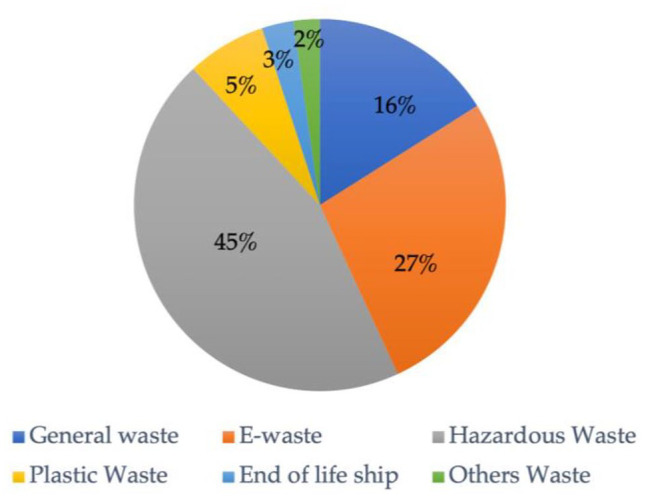
Distribution of attention to the various waste streams^a^ in the academic literature articles published between January 1985 and January 2021 (*n* = 218). ^a^The waste categories are derived from how they were designated in the reviewed literature. E-waste, end-of-life ships, etc., are product-based waste categories, whereas plastic, metal, etc. are material-based. These categories may overlap; for instance, e-waste and ships may contain plastic and metal with various properties and subcategories within them.

**Figure 3.**
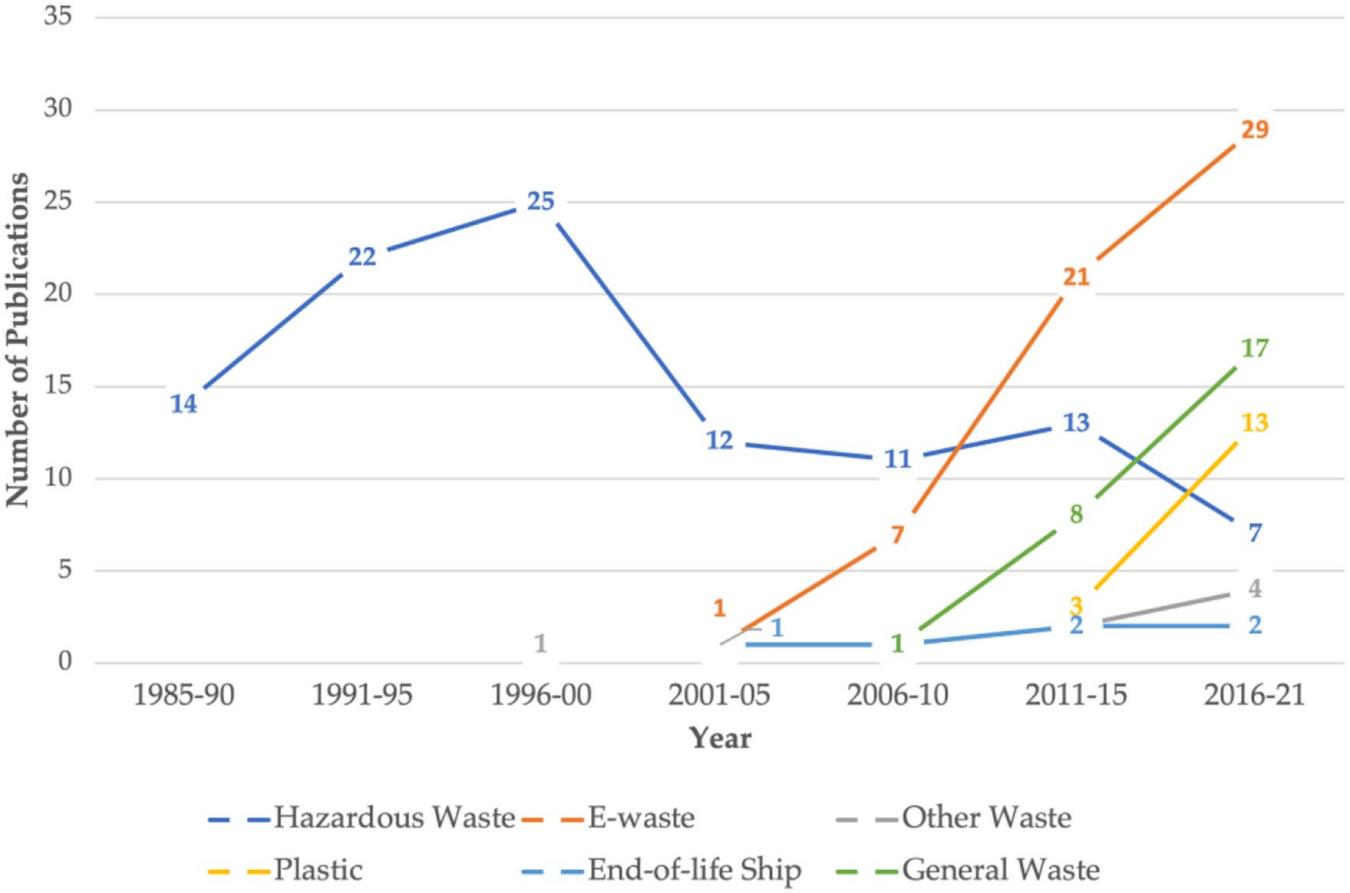
Various waste streams^a^ and the number of academic articles published (*n* = 218). ^a^ *Ibid.*

Between 1985 and 2000, most of the scholarship focused on transboundary *hazardous* waste movement (Figures 2 and 3) and/or the Basel Convention, which governs it. Stricter environmental regulations and increasing prices of environmentally and socially responsible waste management in the Global North often led to toxic waste dumping in countries without stringent laws at a fraction of the cost (Bernard, 2015; Kellenberg, 2012). Some of the many infamous dumping or attempts to dump toxic waste in several Global South countries in the 1980s and early 1990s include an Italian company dumping toxic waste in Nigeria to avoid European regulations, the Italian mafia dumping toxic waste in Lebanon during the civil war when the Lebanese government ceased to function, a U.S. company selling toxic waste mixed with fertiliser to the Bangladeshi government with assistance from Asian Development Bank, and the French government’s deal to dump radioactive and industrial waste in Benin for helping national debt payment at a fraction of the actual disposal cost (Brownell, 2011; Clapp, 2001; Hägerdal, 2019). Several studies (Clapp, 2001; Paul, 2004; Swaney, 1994) cite a leaked 1991 memo from Larry Summers, then chief economist and vice president of the World Bank. He made a case for ‘more migration of the dirty industries to LDCs [Least Developed Countries]’, stating ‘the economic logic behind dumping a load of toxic waste in the lowest wage country is impeccable’ and ‘I’ve always thought that under-populated countries in Africa are vastly under-polluted. . .’. This line of reasoning, described by various scholars as perverse economic rationality, combined with several high-profile cases of toxic waste dumping, led to an increased investigation and scholarship in the transboundary movement of hazardous waste, which has since dominated the field of research (Figure 2). These incidents, termed ‘toxic colonialism’, initially by Greenpeace, afterwards used in academic literature (Clapp, 2001; Kone, 2014; Okereke, 2006), captured the imagination not only of the academia but also of the society at large, leading to the establishment Basel Convention (enforced since 1992) and several other regional conventions and agreements on transboundary movement of hazardous waste.

Around 2000, with increasing societal awareness of e-waste management and regulations in some parts of the world, the academic interest in e-waste increased (Figure 3). E-waste contains valuable substances like gold, silver, copper, etc. Some discarded electrical and electronic equipment can be disassembled to salvage these valuable metals for reuse and recovery. The *Global E-waste Monitor*, published by Global E-waste Statistics Partnership, estimates 53.6 million metric tonnes (mt) of e-waste generated in 2019 is worth an estimated 57 billion dollars, making it the most valuable waste stream, of which merely 17.4% was collected and recycled formally (Forti et al., 2020). E-waste generation globally is up 21% from 2015 and is estimated to increase to 74 mt by 2030 (Forti et al., 2020). E-waste contains toxic elements making its safe management difficult and expensive. Even though e-waste flow between OECD to the non-OECD country is illegal under the Basel Convention, the high disposal cost in high-income countries causes a flow to non-OECD countries (Bisschop, 2012). Its unsafe management causes health and environmental problems (Fazzo et al., 2017; Lipman, 2015). Because of the repair or reuse potential of some discarded electronic goods, e-waste is also disguised as useable or second-hand electronics, sometimes posing as humanitarian donations to circumvent international and national laws on hazardous waste (Bisschop, 2012). For all these reasons, research interest in e-waste has grown significantly since 2000 (see Figures 2 and 3).

Plastic has become another popular transboundary waste stream for research in recent years (Figures 2 and 3). Plastics research has gained momentum due to the rising plastic pollution problem, especially in the marine environment (Borrelle et al., 2020). When China, the biggest importer of plastic waste, stopped importing in 2018, it had a global effect. Since then, the research interest in transboundary plastic waste has been increasing (Figures 2 and 3). Articles that address all waste categories and focus on theory, governance and/or management are an increasing academic research interest and are depicted in Figure 3 in the ‘general waste’ category. Academic interest in transboundary movement of waste categories, such as metal, paper, textile, vehicles, and batteries, remains low (shown as ‘other waste’ in Figure 3) thus far.

This review shows that researchers have often investigated contemporary societal trends of transboundary waste – hazardous waste since the early 1980s, e-waste since the early 2000s and plastic waste since the 2010s. The scholarship in the transboundary movement of waste is increasing as waste generation is projected to increase by 70% compared to 2018 levels by 2050 (World Bank, 2018). However, the analysis shows limited research on other waste streams with transboundary movements such as paper, textile, rubber, wood, metal, batteries, photovoltaic and glass.

### Geographical representation and publication platforms

Academic institutions based in the United States, United Kingdom, China, Canada and Australia were the top five contributors to the transboundary movement of waste literature (see Table 1). The majority (82.11%) of publications were from researchers affiliated with the institutions in OECD countries. In comparison, the non-OECD countries represented only 17.98% of the scholarship, predominantly from the institutions in China (16), Nigeria (4), Taiwan and India (2 each). As determined by the involvement of institutions in two countries or more, cross country collaboration represents only 20% of the total publications. United States (32.40%) and the United Kingdom (13.97%) led among the OECD countries, whereas China (41.03%) led among the non-OECD.

**Table 1.** Number of publications originated from the institutions in various countries (*n* = 218).

OECD (82.11%)	Number of Articles	Non-OECD (17.89%)
United States	58	n/a
United Kingdom	25	n/a
n/a	16	China
Canada	15	n/a
Australia	13	n/a
Japan	10	n/a
Germany	9	n/a
Italy	7	n/a
Switzerland	6	n/a
Belgium, Sweden	5	n/a
Spain	4	Nigeria
Ireland, Netherlands, Norway	3	Taiwan, India
Austria, Greece, Slovenia, South Korea	2	Hong Kong, Thailand, Philippines, South Africa
Czech Republic, Denmark, Finland, France, Portugal	1	Brazil, Croatia, Lebanon, Malaysia, Serbia

In most of the reviewed literature, the non-OECD countries are the recipients of the transboundary movement of waste, yet scholarly contribution from the institutions based there remains low. At first glimpse, this could be attributed to Scopus search limited to the English language. However, Nigeria, India, South Africa and Hong Kong (4, 3, 2 and 2, respectively) all have English as an official language as a legacy of colonialism, which is also a feature of numerous other non-OECD countries. A further explanation for the unequal geographic distribution of publications is likely to be the existing global inequalities, not only in economic conditions and access to higher education but also in the unlevel playing field in academic publishing (see Schipper et al., 2021 for similar inequity in climate scholarship and a manifesto for action). It is worth noting the majority of publications from the institutions in non-OECD countries discusses environmental and social injustice and advocates stricter policies, including the ban on the transboundary movement of waste (Gutierrez, 2014; Kim, 2006; Nnorom and Osibanjo, 2008a; Sonak et al., 2008; Sthiannopkao and Wong, 2013). The highest number of non-OECD country research output is from institutions in China (16), Nigeria (4) and India and Taiwan (3 each). Incidentally, China was the most prominent destination of plastic waste and e-waste import until 2018, and Nigeria is currently one of the biggest e-waste destinations in Africa. This underrepresentation in the research contribution of the countries that also are popular destinations of transboundary movement, and the lack of research collaboration with institutions in these countries could suggest a hegemonic discourse in the field.

The leading journals for publications in the field are *Resources, Conservation and Recycling* (20), *Waste Management and Research* (6), *Environment* (5), *Environment Policy and Law* (5), *Journal of Cleaner Production* (5), *Environmental Law and Policy* (4), *Journal of Environmental Management* (4) and *Waste Management and the Green Economy: Law and Policy* (4). Still, these represent only one-third of the articles reviewed. A wide variety of academic disciplines and sub-disciplines study the transboundary movement of waste, covering a total of 117 different journals. Most publications were from academic institutions of higher education, and only a handful was from not-for-profit organisations and consultancies.

### Data scarcity

Uncertainty quantifying the flows and fate of transboundary waste is one of the most prominent challenges in researching the field (see Bisschop, 2012; Davis and Garb, 2019; Parajuly and Fitzpatrick, 2020 for e-waste; Thomas and Fannin, 2011 for toxic waste, Bishop et al., 2020 for plastic waste). Lepawsky (2016) created a case for data scarcity influencing the discourse by giving an example of how a non-peer-reviewed report, with questionable extrapolated anecdotal data, became the second most cited document in the academic literature for e-waste flows. Thus, extrapolated data on transboundary e-waste flows can be used to push certain worldviews in academia and policymaking (Lepawsky, 2016). Apart from flow, there is insufficient data to show what happens with waste after transboundary movement making it difficult to determine its sustainability impact. Early in the discourse, Schenkel and Skinner (1985) highlighted the need to close the information gap between scientific and technical experts and government and practitioners. Thirty-six years later, these gaps remain and represent obstacles to understanding the transboundary movement of waste.

Several reasons contribute to data scarcity. Firstly, most countries lack robust data monitoring and recording systems, often leading researchers to make ‘best guesstimates’ to understand the phenomenon (Bisschop, 2012). Some countries lack even the basic system for data management (U.N. Environment, 2018). In cases of available data, the quality is limited. For example, most data on transboundary e-waste are collected in weight (and sometimes economic value), which does not tell the properties and other values of the waste (Lepawsky, 2016). Two different compositions of the same weight of identical waste streams, such as plastics, e-waste or construction waste, can have varying properties, toxicity, monetary and socio-economic and environmental values. Secondly, whatever data is collected lacks harmonisation across countries (Bisschop, 2012). This remains a challenge for comparison, even between locations with more sophisticated systems in place. For example, the variety of waste legislation also contributes to the lack of data harmonisation across countries (Lipman, 2015; Obradović et al., 2014). This problem can be seen in the data on waste exports provided by the Basel Convention and UN Comtrade database (Gregson and Crang, 2015), due to reporting gaps by individual countries (Lipman, 2015). Thirdly, various loopholes in the waste legislation (Barsalou and Picard, 2018; Lipman, 2015; Lucier and Gareau, 2016) make manipulation easier and data capture difficult. Illegal transboundary movement makes data gathering difficult, if not impossible (Bisschop, 2012). Fourthly, the engagement of the informal sector, which makes a valuable contribution to waste management (Millington and Lawhon, 2019), makes it difficult to capture data during the value chain of waste management. And lastly, there is a lack of prioritisation for rigorous data. Often port authorities lack the system, human resources, skills, software, language translation skills or funding to capture data (International Criminal Police Organization, 2017).

Given the unreliability of quantitative estimates of waste flows, it may be unsurprising that qualitative studies dominate the literature. Eighty-seven percent of the 218 reviewed articles were qualitative using descriptive analysis. These studies included case studies, policy and legal reviews, historical analyses, text or literature reviews, etc. Quantitative articles (13%) involved mathematical modelling, survey and quantitative material flow analysis. In the reviewed literature, anecdotal data, expert interviews, surveys, estimates and modelling were frequently used to tackle the problems of unreliable data on waste flows. There are notable recent exceptions, which may be a sign of improvement. The *Person in the Port Project* (Odeyingbo et al., 2017), where researchers spent 16 months at two harbours in Lagos, Nigeria, inspecting 3622 import documents, 2184 vehicles and 201 containers in 2015 and 2016, provides some of the most robust data on reusable and end-of-life electric and electronic equipment movement to Nigeria from the rest of the world. Using U.N. Comtrade data, Bishop et al. (2020) showed how the to-be-recycled plastic waste shipment from the European Union to elsewhere creates pathways for plastic debris in the ocean and showed that a significant (between 32,115 and 180,558 tonnes or 1%–7%) of the exported plastic waste was ended in the ocean in 2017. However, similar systematic data-intensive research remains infrequent, meaning that it is still only possible to estimate or guesstimate international movements of waste streams.

### Policy gaps

Using comparative waste policy analysis in the EU and USA, Smith (1993: 91) identified the lack of a standard definition of waste and concluded it was a ‘prerequisite to establishment of workable national and international waste management strategies’. This definition gap is still relevant now. For example, according to the Basel Convention definition, e-waste ‘may or may not be considered waste in general or hazardous waste specifically’, enabling the same e-waste to be hazardous in one country and non-hazardous or even functional equipment in another (Barsalou and Picard, 2018: 899), adding a geographical dimension to the discrepancies that can happen within one country. The export of used electronic and electric equipment, which can extend the lifespan of products, and minimise or even delay waste generation, becomes a loophole for transboundary movement of near end-of-life used equipment and e-waste if the export’s functionality and durability are not guaranteed. Similarly, the export of to-be-recycled waste to countries without sustainable recycling capacities creates a recycling loophole. Despite efforts to devise laws at national, regional and international levels, such loopholes allow the circumvention and manipulation of the law. These loopholes, combined with the high monetary value of waste and the increasing economic cost of environmentally sound management, create incentives for illicit and illegal activities. For e-waste, a high-value waste, the governance often involves actors engaged in white-collar crimes possibly benefiting from these loopholes (Bisschop, 2016).

As 45% of the reviewed literature is on hazardous waste (see Figure 2), the Basel Convention and its Ban amendment remain the most widely discussed policy in the reviewed literature. Various scholars question the effectiveness of the Basel Convention and the Ban amendment (Argüello Moncayo, 2016; Barsalou and Picard, 2018; Gutierrez, 2014; Krueger, 1999; Onzivu, 2013; Reis, 2016). The Basel Convention is the key multilateral international policy that has governed the transboundary movement of hazardous waste since 1992 and is often criticised. When framed, it aimed to regulate the transboundary waste movement of waste from the OECD to non-OECD countries. The Basel Ban Amendment, which makes the flow of hazardous waste intended for final disposal, reuse, recycling and recovery from OECD, EU and Liechtenstein to non-OECD countries illegal, took 24 years to ratify. Analysing the current global flows of e-waste not just limited to OECD to non-OECD, Lepawsky (2015a) questioned the relevancy of Basel’s geographical division of non-OECD and OECD. Reis (2016) called for more dynamic rights-based international law focussing on human, environmental and economic rights. Portas (2016: 79) compared the existing loopholes in the Convention to a tennis racket where ‘there are more holes than matter’ and argued a need for modernisation of the Basel Convention to promote energy efficiency, sound recycling and sustainable material use. Khan (2016a) argued for greater transparency through implementing social labelling system for secondary commodities, adopting Extended Producer Responsibility at the national level, and incorporating sustainable production and consumption instead of merely waste management and cleaner production in the Basel Convention. Parajuly and Fitzpatrick (2020) advocated realistic, comprehensive and integrated policies that focus beyond just economics and considered relevant policies internationally. The slow international political response to the urgent need to halt global flows of hazardous waste led to other international arrangements. The Bamako Convention, effective since 1998, prohibits the import of hazardous waste in Africa. The Stockholm Convention, effective since 2004, limits the transboundary movement of Persistent Organic Pollutants in certain waste. The Rotterdam Convention, effective since 1998, limits the transboundary movement of certain hazardous chemicals. This widening of the scope of the ban has not removed the underlying influences on transboundary waste shipments. Transboundary waste crime continues (Bisschop, 2016; Liddick, 2010; Rucevska et al., 2015; Walters and Loureiro, 2020) and is rising (Andreatta and Favarin, 2020; Dimitris and Alexandra, 2020; INTERPOL, 2020). Existing policies have loopholes that can be and have been manipulated or exploited.

In the cases of a waste stream not covered by the Basel Convention but which follows the WTO guidelines, policies monitoring the social, ecological and economic impacts of such trade remain in the legal grey area (Grosz, 2016). However, there are no international policies to guide the fate of most internationally traded waste (outside the Basel jurisdiction) to maximise the quality of value retention and minimise the associated potential socio-ecological harm (Grosz, 2016).

### Sustainability implications of transboundary waste movement

Waste generation and its transboundary movement relate to the environmental, social and economic dimensions of sustainability. Improper municipal waste management contributes to greenhouse gas emissions (Šomplák et al., 2019). Improper waste management leads to ecosystem contamination and has adverse health effects (Orloff and Falk, 2003; Stebbins, 1993). Transboundary movement of waste to destinations without the capacity for sound management causes harm by creating health problems, especially for people engaging in the informal sector of waste management and the marginalised people in the community (Bisschop, 2012; Fazzo et al., 2017; Lipman, 2015; Okereke, 2006). From this perspective, the transboundary movement of waste is unethical, viewed as exploiting the poor and the most vulnerable, and thus exacerbates inequalities (Little and Lucier, 2017; Orlins and Guan, 2016b). It is portrayed as harm inflicted by one society on another, a case of social injustice and toxic colonialism (Lipman, 2015; Lucier and Gareau, 2016; Müller, 2019). Apart from social injustice, transboundary waste flows are linked to environmental injustice and environmental racism (Clapp, 2001; Iles, 2006; Klenovšek et al., 2011; Lipman, 2006, 2015; Pellow, 2008; Sakai et al., 2011; Temper et al., 2015). These contrasts with earlier views of waste as a resource, an economic opportunity and an argument for a market mechanism to drive the transboundary movement (Alter, 1997; Kofi Asante-Duah et al., 1993; Montgomery, 1995). Kellenberg (2012) showed that waste trade to countries with sound recycling capacities can translate to environmental and economic benefits. China gains economically from imports of wastes such as plastics, cardboard, e-waste, etc. (Ma et al., 2020; Shinkuma and Huong, 2009), and Bangladesh gains economically from importing end-of-life ships (Alam and Faruque, 2014; Paul, 2004) but not without socio-ecological costs. In the absence of holistic, bottom-up solutions to tackle transboundary waste, Davis et al. (2019) showed concerns regarding the further marginalisation of the informal waste sector workers whose livelihood depends on (imported) waste. The transboundary movement of waste is thus closely related to ecology and health, social inequities and injustices, economic and resource benefits, good governance, etc., ultimately tied to various sustainability dimensions.

### Reduction of transboundary movement of waste

Waste is a consequence of economic activity. In the reviewed articles on transboundary waste, consumption is acknowledged as a waste-producing activity that causes transboundary waste movements. Waste literature addresses concepts such as zero waste, cradle to cradle, circular economy, etc., which capture waste reduction. However, there is little discussion on waste reduction in transboundary waste literature. Notwithstanding that one of the objectives of the Basel Convention is to reduce the transboundary movement of hazardous waste, it focuses exclusively on managing the transboundary movement of hazardous waste rather than its reduction (Stebbins, 1993). O’Keefe (1988: 88), one of the earliest to call the transboundary movement of hazardous waste toxic terrorism, wrote, ‘the only solution would be to develop new processes that produce as little waste as possible or failing that, to recycle or neutralise it’. Barsalou and Picard (2018: 890) are sceptical of current environmental conventions leading to waste reduction and argued that they are ‘tools for the global distribution and management of disposable waste’ by commodifying negative externality instead of reducing them. Bisschop (2012) identified the rapid growth and consumption of electric and electronic devices, which produces more e-waste, as a significant push factor for its illegal transboundary movement. Early results from the Chinese ban on plastic imports show environmental gains, especially when the previously exporting countries focus on local management and treatment of plastic waste (Wen et al., 2021). According to prevalent waste hierarchies that prioritise waste prevention, the principle of prevention (Duvic-Paoli, 2018) and proximity principle (Reese, 2018), reducing the volume of transboundary waste by first reducing waste production and then having sustainable value retention, maintenance and recovery options locally seems more sustainable. Reducing transboundary waste needs stewardship rather than management, adding and retaining value instead of trading and international disposing of waste, which appears lacking in the reviewed literature. Similarly, the reviewed academic discourse also lacks discussions on local and regional waste management and its linkages to (reducing) the transboundary movement.

## Analysis and discussion: Transboundary waste binaries

This section establishes the presence and dominance of transboundary waste binaries in the academic discourse, their usefulness and limitations and proposes a nuanced and contextual research approach moving forward. We used Alvesson and Sandberg’s (2011) problematisation approach to spot assumptions and explore their implications. The primary aim of the problematisation method is to ‘re-evaluate existing understandings of phenomena, with a particular view to challenging and reimagining our current ways of thinking about them’ (Alvesson and Sandberg, 2020: 1297). Since understanding waste and its movements is contingent on political, economic, social and cultural contexts, it is essential to look at their underlying assumptions. We spotted and challenged such assumptions to think differently than how and what we already know.

### Waste or resource or both: Contextual and transitional

Thompson (1979) argued that waste is a socially defined construct that moves across the boundary between rubbish and non-rubbish, making the conception of waste contextual and transformational. Thus, transboundary waste can broadly be constructed either as discard or rubbish (something to get rid of) or resource or non-rubbish (something of value) or something in between based on the context. Brownell (2011) argued, ‘as waste became an increasingly complex thing to buy and sell, its definition also became more politically, biologically, and economically contentious’ (p. 264). These transformational and contentious conceptions of waste are compounded by the advent of ideas relating to the circular economy and industrial symbiosis/ecology, whereby waste is increasingly seen as a resource or value to be retained (Deutz and Frostick, 2009; Lacy and Rutqvist, 2015). Such views call to see waste as an input (see McDonough and Braungart, 2002) or even call for an ‘end of waste’ status (enabling a waste material to be legally treated the same as a new equivalent) (Deutz et al., 2020), bringing further contingencies.

The level of socio-political uptake of a circular economy, cradle to cradle, zero waste or many similar concepts can thus influence waste characterisation: either as discard, resource or somewhere in between. However, uptake of these concepts in one country can also have unintended consequences elsewhere. Electronics sent overseas designated for reuse can be diverted as e-waste for profitability in the absence of transnationally harmonised standards for dismantling/recycling and regulatory enforcement (Milovantseva and Fitzpatrick, 2015). Even though polyethylene terephthalate (PET) waste is globally discarded, seeing the economic value despite the ecological cost, China allowed the import of 40% of global plastic discards until recently (Ma et al., 2020). Bangladesh imports discarded end-of-life ships for economic reasons irrespective of the negative environmental and social implications (Alam and Faruque, 2014). Sometimes, the import of discarded second-hand products keeps them from being waste by extending their life elsewhere but might be dumped and landfilled instead of being sustainably managed when it approaches its end-of-life. Importing waste to recycle for secondary materials can also thwart domestic traditions and innovations. Transboundary waste can be an economic ‘resource’ and simultaneously cause socially, politically or environmentally harm even in the same context.

### Transboundary waste binaries informing our understanding and shaping our practices

**Figure 4.**
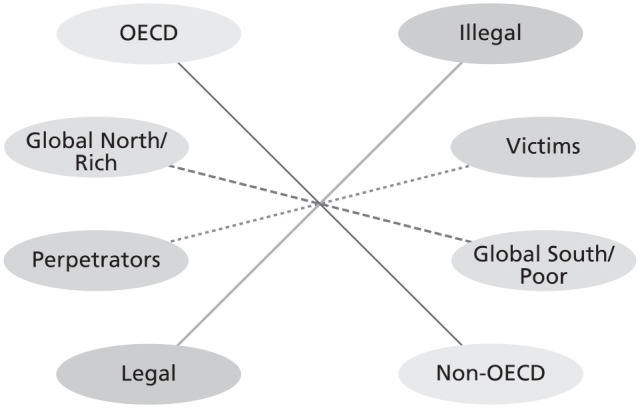
The variety of transboundary waste binaries present in the academic discourse of the transboundary movement of waste, which has roots in seeing waste either as a resource or discarded based on the socio-cultural, economic and political context.

Based on assumptions, we established that waste perception is contextual and can be (harmful) discarded, (useful) resource or in-between. Questioning such assumptions (Alvesson and Sandberg, 2011) in the context of waste fluidity between waste and non-waste (Thompson, 1979) enables the emergence of transboundary waste binaries. Transboundary waste binaries describe the recurrent themes of strong opposing views prevalent in the literature primarily based on assumptions of waste. The most common transboundary waste binaries found in the literature are *victim versus perpetrators, developed/rich versus under-developed/developing/poor* and *Global North versus Global South*. This act of reducing complex interconnected phenomena involving diverse actors and their interests and varied politics into simple opposites represents a simplified approach to understanding the phenomenon. Such binaries, however, do not adequately represent the various contexts and how they interrelate with each other. For example, the Global North is often characterised as a group of rich countries that dump waste onto the Global South for cost-saving reasons despite having the capacity to process waste. This generalisation rightly brought awareness to frequent illegal dumping activities in the 1980s and the 1990s into the academic discourse but is still referred to without newer contextual realities of present times. Research shows such activities are often conducted by actors (legal or illegal) motivated by various push, pull or facilitating factors (Bisschop, 2012), not countries. Many national and international laws and policies make the transboundary movement of hazardous waste illegal. Thus, the prevention of illegal ‘dumping’ and waste is a shared responsibility of Global North and Global South authorities. Relying on such binaries without questioning them can introduce ambiguity in understanding rather than enhancing clarity, which in turn affects implementing potential solutions. The lack of reliable data on the transboundary movement of waste, the criminal nature of the hazardous waste trade and the lack of common definition of waste in the law and policy realm further strengthens these binaries. Some of the more popular transboundary waste binary narratives, either explicitly or implicitly, found in the reviewed literature are listed in Table 2 and later illustrated in Figure 4.

**Table 2.** Various transboundary waste binaries and their implicit or explicit usage in the academic literature ordered from low to high chronologically.

Transboundary waste binaries	Academic Source
Victim versus perpetrators (*n* = 7)	Bisschop and Vande Walle (2013), Brownell (2011), Johnson and Niemeyer (2008), Clapp (1994, 1998, 2002), Kohn (1995)
South versus North (*n* = 15)	Cotta (2020), Millington and Lawhon (2019), Müller (2019), EU (2019), Gregson et al. (2016), Khan (2016), Lucier and Gareau (2016), Bernard (2015), Crang et al. (2013), Marcoux and Urpelainen (2012), Brownell (2011), O’Neill (1998, Paul (2004), Montgomery (1995), Puckett (1994)
Illegal versus legal (*n* = 19)	Bakhiyi et al. (2018), Bisschop (2016), Efthymiou et al. (2016), Orlins and Guan (2016a), Bernard (2015), Kellenberg (2015), Obradović et al. (2014), Lawhon (2013), Bisschop (2012), Liddick (2010), Porter (2010), Benson et al. (2009), Moen (2008), Nnorom and Osibanjo (2008a), Lipman (2006), Soskolne (2001), Montgomery (1995), Sánchez (1994), Hilz and Radka (1991)
Developed (rich) versus Developing (poor) (*n* = 21)	Deshpande et al. (2020), Gollakota et al. (2020), Torrente-Velásquez et al. (2020), Hägerdal (2019), Müller (2019), Bisschop (2016), Lucier and Gareau (2016), Nnorom and Osibanjo (2008b), Okereke (2006), Yu-Jose (2004), Tsimplis (2001), Alter (2000), Krueger (1999), Ryland (1999), McKee (1996), Sánchez (1994), Kummer (1992), Anyinam (1991), Lipman (1990), Kohl and Sud (1989), Wynne (1989)

#### Victim versus perpetrators transboundary waste binaries

In the context of the socio-ecological justice discourse in the social sciences, *victim versus perpetrators* is one of the transboundary waste binaries that appear implicitly or explicitly following the cases of hazardous waste dump from OECD to non-OECD countries ( see examples discussed in ‘Trends and waste categories’ Clapp, 1994; Favarin and Aziani, 2020; O’Keefe, 1988). This binary goes beyond hazardous waste and applies to other waste streams like e-waste, plastic and end-of-life ship, often associated with health and environmental harm if the destination country cannot manage imported waste well (Cotta, 2020). In this binary, waste is predominantly seen as discard and is ‘discarded to’ destinations without infrastructures and capacities. The social and environmental risks and burdens associated with the transboundary waste movement are distributed unequally, thus characterising the Global North as the perpetrators and Global South as the victims (Cotta, 2020; Favarin and Aziani, 2020). Using a quantitative model, Favarin and Aziani (2020: 372) confirmed their hypothesis that ‘countries sharing a colonial relationship are more likely to exchange illicit waste’. This binary is motivated to showcase disregard for equity, fairness and distributive justice in the phenomenon of the transboundary waste movement and is characterised by toxic colonialism, garbage imperialism and environmental racism (Brownell, 2011; Lipman, 2015; Müller, 2019; Okafor-Yarwood and Adewumi, 2020).

The victim versus perpetrators binary is also present in environmental criminology. It is associated with fraudulent or illegal behaviour, where bad actors take advantage of legal and policy loopholes and cause socio-ecological harm. U.N. Environment (2018) estimates environmental crimes, including the transboundary movement of waste, as the fourth largest and most lucrative crimes after drugs, counterfeits and human trafficking. It further identifies most waste trafficking to originate in the so-called ‘developed countries’ and is driven by financial gains, weak enforcement systems and complexities in actors and policies (U.N. Environment, 2018). By sending discards as recyclables, e-waste as used equipment, mixing discards in recyclables, etc., the perpetrators create victims driven by economic gains. While this transboundary waste binary highlights the socio-ecological injustices, it fails to interrogate the perpetrators and their actions in context. Global North as the predator is the underlying assumption, which can be challenged. While Global South remains unequal and global inequality is increasing due to systemic problems (Hickel, 2017), Global South countries also have sovereignty and agency to refuse waste coming from Global North and, in many cases, protected by international and national laws.

#### Legal versus illegal transboundary waste binaries

The contingent definition of waste and lack of harmonisation of laws and policies across jurisdictions create various loopholes and a thin line between legal and illegal transboundary waste. In a context where nations prohibit the trading of certain waste but liberalise others, Khan (2016) argued that the blurring between legal visions of waste and commodities is inevitable and confusing as commodities turn to waste and increasingly waste turns back into commodities. Bisschop (2012) identified actors and their varied relationships, global asymmetries in law, culture and economics and unharmonised policies to make a case for a thin line between legal and illegal in the worldwide movement of e-waste. Such a fine line can be crossed during waste transportation or during collection or disposal (Bisschop, 2016). Unlike other transboundary waste binaries, the law attempts to define legal/illegal yet remains prone to varied interpretations. Given the diversity of actors, their intentions, various stages and interpretation of the law, the legal–illegal binary shares the characteristic of being porous. International laws remain vague, and their interpretation is often shaped by socio-economic context. For example: used electronic and electric equipment is legal, whereas e-waste is illegal to ship from OECD to non-OECD countries, but what e-waste constitutes also depends on the socio-economic context such as the ability and capacity of authorities to check for functionality. As environmental crimes become increasingly lucrative financially (Walters, 2013), the ambiguous assumptions between legal and illegal transboundary waste movements need more context and clarity.

#### Global North versus Global South transboundary waste binaries

*Global North versus Global South* is another popular transboundary waste binary. This binary focuses on the economic, social and geographic distinctions. It also captures a similar discussion in the (*Developed versus Developing*) and *Rich countries versus Poor countries* binary. Assumptions of geographies of waste movement in this binary are limited to flow from the North to the South and fail to consider other directional movements. Actors from the Global South, whose coordination facilitates such North–South movements, are not discussed. Quantitative research on e-waste shows illegal e-waste flow from higher-income countries to lower-income countries (Efthymiou et al., 2016). A 2-year study in Nigeria at the two import ports showed used electronic and electric (UEEE) devices imported in containers and roll-on/roll-off vehicles originating from the EU, USA and China, of which 26% of the total 60,000 tonnes were estimated to be e-waste (Odeyingbo et al., 2017). Such UEEE flows were confirmed by follow-up research in Ireland, with 17,319 kg of UEEE exported on roll-on/roll-off vehicles (McMahon et al., 2021). Based on the customs paperwork, the Nigerian research shows that most of the registered UEEE importers were Nigerians (Odeyingbo et al., 2017). Other research shows waste flow other than the North–South direction, showing countries in Africa as exporters of e-waste elsewhere (Lepawsky, 2015a), thus challenging the assumptions behind North–South flows.

Analysing the global PET plastic trade case study, Furniss (2015) showed similar assumptions and proposed reframing the transboundary waste movement beyond the North–South directionality. Limited data and unreliability of existing data limit our ability to judge the veracity of assumptions in this binary. Some recent research challenges inherent assumptions and shows how adhering to North versus South binaries limits our understanding, which then can creep into the policy and action realm.

#### OCED versus non-OCED transboundary waste binaries

The binary of OECD and non-OECD represents the international waste governance perspective and is present in the Basel Convention, especially in its Ban Amendment. Ban Amendment aimed to ban the movement of hazardous waste from OECD to non-OECD countries, but not flows from non-OECD to non-OECD or non-OECD to OECD countries. The transboundary waste binary assumes that all countries grouped in one of the two categories have the same waste governance and management capacity and fails to see the diversity within the groups. Such assumptions distort our understanding and limit effective policymaking. The pollution haven hypothesis predicts the pollution-intensive transboundary movement of waste flow to destinations with low environmental standards. It can also be traced back to the rich or OECD versus poor or non-OECD binary, where the former have sophisticated environmental health and safety standards. But when examined empirically, this hypothesis fails to capture the full context and complexities of the phenomenon (see Crang et al., 2013; Davis et al., 2019; Lepawsky, 2015a, 2015b; Moore et al., 2018).

Crang et al. (2013) highlighted that waste flows are influenced by supply and demand in global value chain networks and do not necessarily follow OECD to non-OECD or Global North to Global South. Both Davis et al. (2019) and Moore et al. (2018) suggested a shift of waste flow analysis from the macro scale to the micro to incorporate local communities and context, realities, opportunities, risks and vulnerabilities. Context-sensitive research challenges the dominating geographic assumptions of transboundary waste flows and shows limitations to assumptions behind these binaries.

### Limitations of transboundary waste binaries narratives

The transboundary waste binaries in the literature demonstrate the unequal global context and the social and environmental injustices that arise from the transboundary waste movement. However, some binaries come with questionable assumptions, as discussed earlier. Thus, the widespread usage of these transboundary waste binaries, unless relevant from more contextual and empirical analysis, limits problem-solving discourses from the literature. Instead, these binaries, limited to problem identification and description, fail to capture the complexity of the global sustainability challenge of transboundary waste movement, whose socio-ecological impacts are not only limited within the narrow transboundary waste binaries imaginary of rich versus poor, North versus South, etc. but also the whole earth, humanity, present and future generations. Assumptions that form these binaries further restrict effective policymaking. A more nuanced and contextual approach challenges assumptions and shows limitations in these transboundary waste binaries. The following section highlights some research work that either challenges the existing binaries or adds context to these binaries.

### Looking past the transboundary waste binaries: A nuanced understanding

Some research contributes to the body of knowledge by incorporating social, economic, cultural, historical and political contexts. In the literature, these contributions stand out as bringing nuanced approaches to understanding the transboundary waste movement and challenge assumptions of existing literature by adding layers of relevant context to the research. For instance, Millington and Lawhon (2019) proposed new epistemologies for research on the role of materiality and technology for insights into the distribution of costs and benefits. Their context-sensitive research agenda focuses on relational understanding to explore what enables and constrains value extraction for different actors in the waste value chain. Giving examples of compartmentalised outlooks on waste, Barsalou and Picard (2018) proposed a legal framework of waste on a global scale that looks past the OECD (rich) and non-OECD (poor). They suggested that international laws should go beyond protecting the environment and be a guide to ‘manage and externalise waste on a global scale’ across international law (p. 905). Davis et al. (2019) proposed empirical research to guide theory and policy. They argued that its relevance not only for data richness but contextual political narratives and their policy implications. Müller (2019) advocated for connecting the existing macro-level discussions, characterised by often necessary generalisation on harm and toxic capitalism, with the micro-level focusing on actors and their environment. This author advocated for future research that ‘aims to reinsert the human element while moving beyond anecdotal evidence and to provide personal insights into the business world of those trading’ Müller (2019: 56). Barnes (2019) proposed research on long-term solutions with a mix of innovation, recycling and reducing production and consumption to prevent the tragedy of the commons. Moore et al. (2018) identified the disconnection of the health and environmental risk borne by locals with the nation-state discourse and advocated future research de-centring from the nation-state to the cosmopolitan notion of justice. Torrente-Velásquez et al. (2020) and Wilts et al. (2011) put a case for a global extended producer responsibility system for sustainably managing waste beyond the narrow geographical binaries discussed previously. Temper et al. (2015) called for understanding through collaboration and engaged research between academia and civil society. The researchers investigated multidimensional contexts, challenged existing geographic imaginaries and incorporated politicisation. The diversity of their research focus ranges from technology, social, legal, politics and governance, business and economy and engaged research. These research approaches seek contextual understanding and challenge inherent assumptions to solve our shared sustainable challenges.

## Conclusions and recommendations

This literature review spanning 36 years demonstrates that sustainable waste management and its transboundary movement remain challenging at the local, national and international levels. Bakhiyi et al.’s (2018) comparison of e-waste management practices to ‘opening Pandora’s box’ filled with unpredictabilities and complexities is equally true in the transboundary waste context. Our review reveals diverse characterisations of the phenomenon often reduced to simple and opposing binaries. Depending on varied social, ecological, economic, political and cultural contexts, the binaries are predicated on the assumptions of waste stemming primarily as a resource or discard. Such simplification might serve our understanding but also can restrict or limit it. Lack of data and policy gaps underlie and reproduce these limitations. We argued for a need to look beyond binaries and instead call for a nuanced and contextual understanding of the issues underlying transboundary waste movements. We gave examples of research enabling a more contextual understanding, questioned the assumptions and showcased limitations in the binaries. Furthermore, the literature reviewed shows that the discussion is dominated by managing the transboundary waste movement but pays little attention to reducing it.

The transboundary movements of waste have substantial implications in all three dimensions of sustainability. Apart from the three sustainability dimensions of people, planet and prosperity (PPP), contexts of time and place are necessary for a nuanced and integrated approach (Vermeulen, 2018). With increasing waste generation, there is a need for a sustainable, ethical, just and impactful solution within the planetary and social boundaries. Being mindful of PPP, time and space dimensions and based on the findings, prevalence of transboundary waste binaries and their limitations, we propose five interconnected areas of future research.

Firstly, we propose more collaborative research that brings academic disciplines, stakeholders and their contexts together. Geographical collaboration between the exporters and the recipient or importers of transboundary waste can bring an integrated and life cycle understanding of transboundary waste. Such understanding can inevitably bring thoughts and ideas together across diverse socio-cultural and economic contexts to create inclusive understandings.

Secondly, we propose future problem-solving research, engaging various academic disciplines with society. One option is transdisciplinary research, which is increasingly recognised as a contextual solution-oriented approach to sustainability science. A transdisciplinary approach can bring together the informal waste sector with the formal, inspection workers, lawmakers, recyclers and other actors across countries in an equal participatory process to create problem-solving knowledge. This plurality of ideas can challenge assumptions behind transboundary waste binaries. Irrespective of the location and roles, waste indiscriminately affects the marginalised; their active voices are rare in the research field thus far, despite their service in waste management. Just transition focused research can start with giving them equal and empowering voices in future research.

Thirdly, the lack of reliable data, especially quantifying flows, and the fate of waste, limits our understanding of transboundary waste movement. Research in developing data capture and harmonisation methodologies at a local and international level that also enhances data transparency and availability are essential. Accurate data can give insights into less discussed waste flows within Global South, Global North or from South to North.

Fourthly, we recommend research on preventing transboundary waste movement in the first place. This also includes reducing the generation of waste that is destined for transboundary movement. Strategies can include reducing or refusing consumption and value creation, addition, retention of waste locally and regionally. Such sufficiency approaches are increasingly advocated by emerging research in degrowth, post-growth and the sufficiency-based circular economy narratives.

Lastly, various finance, policy, regulation and law instruments need reform and rethinking to make them effective. However, research should also focus on making these instruments ethical and fair socially and ecologically.

## Notes

1. The Global North and South terminology stems from Brandt’s (1980) map highlighting that the world’s richer countries are largely, but not entirely, in the northern hemisphere. The Global North, thus, includes high-income southern hemisphere countries such as Australia and New Zealand. Notwithstanding the outstanding rates of economic development in parts of the world since 1980 (most notably China), the division retains validity. Although the Global North does not have a fixed membership in the sense of the OECD, both are largely economic in definition and consequently broadly similar in composition.
